# Recognizing the True Value of Testosterone Therapy in Health Care

**DOI:** 10.1089/andro.2022.0021

**Published:** 2022-12-28

**Authors:** Abraham Morgentaler, Abdulmaged Traish, Rajat S. Barua, Paresh Dandona, Sandeep Dhindsa, Mohit Khera, Farid Saad

**Affiliations:** ^1^ Department of Surgery (Urology), Beth Israel Deaconess Medical Center, Harvard Medical School, Boston, Massachusetts, USA.; Departments of ^2^Urology and Biochemistry, Boston University School of Medicine, Boston, Massachusetts, USA.; ^3^ Medical Affairs, Bayer AG, Leverkusen, Germany.; ^4^ Department of Cardiology, Kansas City VA Medical Center, Kansas City, Missouri, USA.; ^5^ Division of Endocrinology, Diabetes and Metabolism, State University of New York at Buffalo, Buffalo, New York, USA.; ^6^ Division of Endocrinology, Diabetes and Metabolism, Saint Louis University, St. Louis, Missouri, USA.; ^7^ Scott Department of Urology, Baylor College of Medicine, Houston, Texas, USA.; ^8^ Medical Affairs, Bayer AG, Berlin, Germany.

## Abstract

There has been little recognition within the medical community of the health impact of testosterone (T) deficiency (TD), also known as hypogonadism, and the substantial benefits of testosterone therapy (TTh) on health and quality of life despite high-level clinical evidence. In a roundtable symposium, investigators summarized the contemporary evidence in several key clinical areas. TD negatively impacts human health and quality of life and is associated with increased mortality. Several studies have demonstrated that TTh in men with TD reduced all-cause and cardiovascular mortality. The longstanding belief that TTh is associated with increased prostate cancer (PCa) risk is contradicted by recent evidence, including multiple studies showing that TTh is associated with reduced PCa risk. Similarly, the weight of current evidence indicates the purported concern that TTh is associated with increased cardiovascular risk is incorrect. Normalization of physiological T reduces myocardial infarction, stroke, and deaths compared with men whose testosterone levels failed to normalize. In diabetic men TTh improves insulin resistance, and a large 2-year controlled study in men with abnormal glucose tolerance showed a substantially reduced rate of diabetes among men treated with TTh compared with untreated controls. Long-term TTh in diabetic men resulted in progressive improvements in obesity and insulin requirements, including a substantial number who experienced complete remission of diabetes. Finally, TTh has been shown to reduce severe outcomes with Covid-19 infection. These lines of evidence argue strongly for the need for greater awareness in the medical community of the impact of TD on health, and of the health benefits of TTh.

## Introduction

Despite high-level evidence demonstrating the health risks of testosterone (T) deficiency (TD, also known as hypogonadism) in men and the substantial benefits of testosterone therapy (TTh), there has been little recognition of the true value of TTh in medicine. The purpose of this article is to highlight several key topics that together provide a powerful argument that TD and TTh merit greater awareness in the medical community.

Testosterone is a ubiquitous molecule among vertebrates, leading to tens of thousands of research studies exploring its effects in animals. The effects of castration on muscle, fat, and sexual behavior have been recognized in humans and domesticated animal species for millennia.^1^ The androgen receptor, a key molecule that mediates the effects of androgens in most human tissues, has been identified in a remarkably broad range of tissues, including but not limited to muscle, bone, bone marrow, the peripheral and central nervous system, adipocytes, liver, kidney, skin, and testis.^2^ No wonder, then, that a deficiency of testosterone has been shown to have numerous negative effects in men, which may be improved or reversed with normalization of serum testosterone through TTh.

Although health care providers (HCPs) may have a general awareness that testosterone is important for male sexual desire and muscle mass, it is not widely appreciated that (1) TD is associated with several of the most important general medical conditions facing our society, including type 2 diabetes mellitus (T2DM), obesity, osteoporosis, and cardiovascular (CV) disease, and (2) TTh has shown compelling health benefits for those conditions, among others. Testosterone levels have been associated with the most urgent global medical issue of the past several years, namely COVID-19 infection,^3^ and TTh has even been shown to reduce the rate of severe infection.^4^ Mortality has been shown to be associated with low levels of testosterone,^5^ and observational studies have shown a twofold decrease in mortality among men with TD that received TTh.^6,7^

Despite these impressive results, it is curious that few HCPs, medical researchers, or health administrators are aware of these potential benefits of TTh. This lack of recognition represents a troubling blind spot in academic medicine. Indeed, correcting this deficiency has the potential to improve health for millions of men in the United States alone, and to save billions of dollars in health care costs.

## The Most Misunderstood Molecule in Medicine

Academic medicine and professional specialty medical organizations place tremendous value on high-quality research studies to create clinical guidelines and recommendations. Yet this open-minded approach to new and evolving information seems to not apply to studies involving TTh.

In the pages that follow are summaries of evidence showing that TTh has important beneficial effects on several of the most common and costly medical conditions, including metabolic disease and obesity, T2DM, and CV disease. Many of these studies have been published in high-impact medical journals by highly regarded investigators. However, these studies are rarely cited in clinical guidelines, with many neglecting even to mention TTh as a possible option.

In 2016, the initial results of the Testosterone Trials were published in the New England Journal of Medicine,^8^ and additional results were published subsequently in specialty journals. At the time, this was the largest placebo-controlled TTh trial to date, involving 790 men ≥65 years with unequivocally low serum T concentrations, treated for 1 year with testosterone gel or placebo gel. Benefits of TTh versus placebo were demonstrated in multiple areas, including not just the expected sexual symptoms such as libido and erections, but also physical activity, self-reported sense of increased energy, mood, bone density and bone strength, and resolution of unexplained anemia. Adverse events were similar in both groups. Despite these remarkable results, the study authors^8^ as well as authors from the U.S. Food and Drug Administration (FDA)^9^ were dismissive, describing the benefits as minor and unlikely to be of clinical benefit.

Patient experiences contradict that assessment. In one study, men with advanced prostate cancer and symptomatic from TD were so desirous of TTh that they were willing to undergo treatment despite being told TTh was likely to cause rapid progression and death (22 pts), and were so pleased with their result that they remained on treatment for a median of 12 months.^10^

What explains this apparent bias against TTh? The most likely answer is an unflattering long-standing narrative regarding testosterone that permeates the general media as well as in medical literature.

The public associates testosterone with bodybuilders, cheating athletes, and toxic masculinity. In medicine, Nobel Prize winner Charles Huggins wrote in 1941 that testosterone “activates” prostate cancer, and this belief persists to the present day despite overwhelming evidence to the contrary,^11^ as reviewed hereunder.

In 2013, an observational study gained international media attention when it reported that men who had received a prescription for TTh had greater risk of heart attack, stroke, and death than men who did not receive TTh.^12^ Although this study was subsequently found to be tainted by the inclusion of 9% females in an all-male study (reviewed in Refs.^13,14^), the reputational damage was done, even though a review of 23 subsequent studies through 2017 found no supporting evidence of increased CV risk.^14^ Many HCPs believe the evidence has shown that TTh is associated with CV risks, even though the weight of evidence strongly suggests normalization of serum testosterone is beneficial for heart disease, as reviewed hereunder.

In this study, we provide summaries of selected key areas of testosterone research to provide a scientific basis for the argument that TD is an important health risk and TTh offers valuable health care benefits with a reassuring safety profile.

## Testosterone Deficiency Is a Serious Health Concern and TTh Improves Signs and Symptoms Associated with TD

Testosterone is a metabolic, sexual, and vascular hormone that plays an important metabolic function in many organs, and plays a key role in human physiology and health. A deficiency of testosterone has been shown to have detrimental effects on men's health and to negatively impact quality of life.^15^ The signs and symptoms of TD encompass sexual, physical, psychological, and cognitive domains.

A large body of evidence has emerged that demonstrates the therapeutic value of TTh in men with TD. The Testosterone Trials^8^ and the Testosterone for Diabetes Mellitus Trial (T4DM)^16^ provided solid foundation of evidence that TTh in older men produce significant improvement in sexual function, mood, severity of depressive symptoms, ameliorates anemia of known and unknown causes, improves bone mineral density and strength, and prevents progression of prediabetes to frank diabetes and is associated with remission of T2DM. TTh is associated with reduced risk of overall and vascular mortality.^17^ Low T is associated with increased incidences of dementia and Alzheimer's disease.^18^

Given the number and scope of new studies showing evidence of clinical benefit of TTh with a very acceptable safety profile, it is difficult to understand the opposition of the FDA to the use of TTh in men without anatomic or selected known causes of TD, which they term “age-related” hypogonadism. There is no scientific basis for the belief that a man with a known cause of TD merits treatment but a man without a known cause does not, since in both cases the problem is a deficiency of the same molecule, that is, testosterone. Nor is there a reason to believe that the risks of TTh are greater for men with age-related TD than men with “classical” TD. In addition, since cases of classical TD are quite infrequent, the subjects in all moderate to large research studies comprise almost entirely men with age-related TD. For these reasons, we disagree with the FDA position on TTh in age-related TD.

## TTh May Be Protective and Therapeutic for Prostate Cancer

Since the early 1940s it was believed that TTh increased the risk of prostate cancer. In fact, many clinicians were taught that TTh was similar to “adding fuel to the fire” to prostate cancer. It was not until the turn of the century when this paradigm began to shift. For the past 20 years, our beliefs of TTh have shifted from TTh being dangerous, to being safe, to being potentially therapeutic, and now to being potentially protective against prostate cancer.

Several studies have demonstrated the potential protective effects of TTh in men with a history of prostate cancer. Pastuszak et al published a series of 103 hypogonadal men receiving TTh, of which 26 men were considered high risk.^19^ High risk was defined as having positive surgical margins, positive lymph nodes, or positive surgical margins. There were 49 eugonadal controls, of which 15 men were high risk. There were significantly fewer biochemical recurrences in men receiving TTh than those men not receiving TTh (15.3% vs. 53.3%, respectively).

In a study involving a national prostate cancer registry, Loeb et al found that patients who received TTh had a lower risk of aggressive prostate cancer (odds ratio [OR], 0.50; 95% confidence interval [CI], 0.37–0.67).^20^ Finally, Ahlering et al found that in hypogonadal men after radical prostatectomy, cancer recurred in only 7.2% of patients treated with TTh, whereas cancer recurred in 12.6% of men who did not receive TTh.^21^ Recurrence rates were 54% lower for men who received TTh. Finally, in men destined to recur, TTh delayed the time to recurrence by an average of 1.5 years.

In a similar vein, a provocative new study investigated the association of TTh use with or without additional use of metformin on hormone-associated cancers, namely, prostate, colorectal, and male breast cancer among older U.S. men.^22^ With TTh alone, the OR for prostate cancer incidence was reduced by one third (OR 0.63, 0.56–0.71). The combination of TTh plus metformin use reduced the incidence by more than 50% (OR 0.44, 0.36–0.54).

The most fascinating studies regarding the therapeutic effects of TTh are those involving bipolar androgen therapy (BAT) to treat metastatic castrate-resistant prostate cancer. In a study by Schweizer et al, 14 of these men were treated with high-dose testosterone injections every 4 weeks while on anti-androgen therapy, resulting in supraphysiological testosterone concentrations followed by castrate testosterone concentrations as the testosterone injection wore off.^23^ Treatment with BAT demonstrated a 50% reduction in PSA and 50% improvement in radiographic response of the metastatic prostate cancer.

In a more recent study known as the TRANSFORMER trial, these authors conducted a randomized phase II study comparing BAT versus standard treatment with enzalutamide—a potent agent that interferes with androgen action—in asymptomatic men with castrate-resistant metastatic prostate cancer.^24^ They found that BAT demonstrated similar time to progression and prostate-specific antigen response. In addition, patients receiving BAT reported significantly greater improvement in quality of life, especially fatigue and sexual function.

Although for decades it was believed that TTh increased the risk of developing prostate cancer, we have now entered an era where TTh is being used in clinical trials for potential therapeutic and protective effects. Although more studies are needed to better understand this protective and therapeutic relationship between TTh and prostate cancer, it is now evident that prostate cancer can no longer be considered a risk of TTh.

## TTh Is a Potentially Valuable Treatment in T2DM

In a study of 103 men with T2DM, 33% were found to have TD.^25^ These men had low total and free testosterone concentrations, along with inappropriately low or normal concentrations of luteinizing hormone and follicle-stimulating hormone, categorizing them as having hypogonadotropic hypogonadism (HH). Testosterone concentrations were found to have an inverse relationship with BMI but were not related to glycated hemoglobin (HbA1c) or the duration of diabetes. Nondiabetic obese men have a prevalence of HH in 25%.^26^ Thus, this is the most common cause of hypogonadism in the community. A study in severely obese young males between 14 and 20 years of age revealed HH in 75% of them. After bariatric surgery, testosterone levels normalized in the majority after 2 years and were maintained in those who maintained their weight loss but diminished again if they regained weight.^27^ Thus, body weight is a major determinant of plasma testosterone concentrations.

It has also been demonstrated that T2DM patients with HH have increased insulin resistance when compared with those without.^28^ TTh restores insulin sensitivity and enhances genes in the insulin signaling pathway, whereas simultaneously reducing adiposity and increasing muscle mass.^15^ Consistent with these observations, TTh has been shown to prevent the development of T2DM. In a randomized placebo-controlled trial, obese men with impaired glucose tolerance or early diabetes, and with testosterone concentrations <400 ng/dL were treated with intramuscular injections of testosterone or placebo for 2 years, in addition to lifestyle changes.^16^ There was a 41% reduction in incidence of T2DM in the testosterone arm. Among the early T2DM patients, there was a reversal of diabetes in 45.2% in the testosterone arm and only 32.1% in the control arm. Tempering these results was an observation of increased hematocrit in ∼25% of men receiving TTh, although only 6% had two such readings, justifying study withdrawal.

Clearly, therefore, testosterone should be measured in all type 2 diabetes and obese patients. If testosterone concentrations are found to be low, TTh has to be considered not only for its beneficial effects on sexual function but also for its metabolic effects that are profound and widespread. Recognition of the value of TTh in diabetes is evolving—one major professional society recommends its use when TD is present, whereas a second advocates testing for testosterone in men with symptoms of TD but does not yet recommend treatment.^29,30^

## Long-Term Benefits of TTh

Most randomized controlled trials involving TTh have been of short duration. In one meta-analysis, one third of the studies had a duration of 3 months or less. Still, the meta-analysis showed robust effects of TTh on reduction in fat mass, increase in lean mass, improved fasting glucose and insulin resistance.^31^

To investigate the long-term effects of TTh, real-world evidence (RWE) is required. An ongoing prospective observational study in a urological office in Germany was initiated in 2004. Patients are routinely screened for TD and if present, they decide whether or not to undergo treatment with TTh after receiving information on the potential benefits and risks.^32^ A subgroup analysis of hypogonadal men with T2DM and obesity with 13 years follow-up was presented at the annual congress of the American Diabetes Association. There were 190 men in the TTh group and 180 men in the control group, all of whom received standard diabetes treatment, including lifestyle modification courses at the local diabetes center.

In the TTh group, men progressively lost weight resulting in a 22% weight loss after 13 years.^33^ HbA1c dropped from 9.5% to 5.5%. Daily insulin dose in patients receiving insulin at baseline was reduced from 38.0 to 4.1 U/d. Remission of T2DM was observed in 56.8% of the TTh group, defined as discontinuation of all diabetes medications and HbA1c <6.5%. In the control group weight increased and glycemic control worsened.^34^

Adverse CV events were greater in the control group. In this group, 23.9% of men died of a CV event, 34.4% had a nonfatal myocardial infarction (MI), and 28.3% had a nonfatal stroke. No patient in the control group died of a CV event or suffered an MI or stroke.^35^

In this long-term RWE registry, TTh for 13 years resulted in substantial and sustainable weight loss and profound improvement of T2DM, with more than half of these patients achieving remission, and with lower rate of CV mortality, MI, and stroke.

## Normalization of Testosterone in Men Is Associated with Reduced Heart Disease

Testosterone exerts a multitude of effects on CV physiology. At physiological levels, T increases coronary vasodilatation and coronary blood flow, improves vascular reactivity, and shortens corrected QT interval. All these effects likely play a role in the mitigation of CV risk.^36^ Indeed, observational studies demonstrated that low T levels were associated with poor clinical CV outcomes such as increased mortality and MI.^36,37^ However, the role of TTh has been controversial, as several studies have suggested poor CV outcomes and adverse effects with TTh.^35,36^ However, no clinical trials of TTh published to date have been adequately powered to assess CV events. Furthermore, published studies have not specifically evaluated whether TTh resulted in normalization of T levels.

To address this issue, an investigation was performed of 83,010 men with TD and without prior history of MI or stroke. Results showed that normalization of serum T levels with TTh was associated with a reduction in all-cause mortality, MI, and stroke compared with men whose serum T concentrations failed to normalize.^37^ In a separate study, it was found that even in men with prior MI there was a decrease in all-cause mortality with normalization of T levels compared with men whose T concentrations remained low, with no increase in recurrent MI.^38^

A recent meta-analysis of 35 placebo-controlled TTh trials involving 5601 men with low baseline T concentrations (≤350 ng/dL) with a mean duration of treatment of 9.5 months found no significantly increased risk of CV events between the TTh group and placebo groups.^39^ An additional study showed that active cigarette smoking nullified these beneficial effects of T normalization with TTh.^40^

Normalization of serum T in men with TD appears to have additional CV benefits. Atrial fibrillation (AF) is the most common cardiac dysrhythmia, associated with significant morbidity and mortality. In a study cohort of 76, 639 men with low T levels it was found that normalization of T concentrations with TTh was associated with a significant decrease in the incidence of AF.^41^

In summary, the scientific evidence indicates that low T concentrations are associated with increased mortality and CV events. In men with TD without prior CV events (MI, stroke, and AF), normalization of serum T with TTh is associated with decreased morality, MI, stroke, and AF. Ongoing cigarette smoking nullifies the MI benefit of normalization of T levels. In men with TD with previous MI, normalization of T with TTh is associated with decreased mortality and is not associated with greater risk of MI.^40^

## TTh Is Associated with Reduced Severe Outcomes from COVID-19

Since the start of the COVID-19 pandemic it has been consistently noticed that patients hospitalized with COVID-19 are more likely to be men than women.^42^ As such, it was presumed that the male sex hormone, testosterone, is a risk factor for severe COVID-19 infections.^43^ However, there is wide variability of serum testosterone concentrations among men.^44,45^ Aging and the presence of comorbidities, which are themselves risk factors for hospitalization from COVID-19, are also associated with TD. Men with chronically low testosterone have weaker muscle mass and strength, which contribute to reduced lung capacity.^46^ This raises the question whether TD is a risk factor for severe COVID-19 illness.

A retrospective cohort study has recently evaluated hospitalizations from COVID-19 in eugonadal and hypogonadal men.^4^ The study included 116 untreated hypogonadal men, 427 eugonadal men, and 180 men receiving TTh. Hypogonadal men had 2.4 times greater odds of requiring hospitalization after contracting COVID-19 compared with eugonadal men. This effect was independent of other known risk factors for hospitalization, including age, comorbidities, immunosuppression, or body weight. In contrast, the odds of hospitalization were similar for men on TTh and eugonadal men. In other words, men with TD on appropriate TTh had a similar rate of hospitalization as men with normal testosterone concentrations, and substantially lower rate of hospitalization compared with untreated men with TD.

Importantly, this risk reduction was limited to men who achieved normalization of testosterone concentrations while on TTh. Men with TD who failed to normalize serum testosterone concentrations despite receiving TTh had 3.5 times greater odds of hospitalization than those on adequate TTh.

These results indicate that (1) TD in men should be considered a risk factor for severe COVID, and (2) prospective clinical trials are needed to explore the efficacy of TTh in preventing hospitalizations after COVID-19 and similar respiratory illnesses in men with TD.

## Discussion

In this article, we have provided brief selected reviews of several key topics related to TD or TTh. In several instances the reviews focus on research performed by one or more of the authors themselves. The information provided in this study amply demonstrates the health impacts of TD and the multiple benefits of TTh across a range of critical health conditions, including T2DM, obesity, CV disease, and COVID-19 infection. In addition, the evidence presented in this study provides reassuring data regarding TTh safety, addressing in particular the two most common concerns, namely the risks of prostate cancer and CV disease. Interestingly, current evidence suggests that TTh in men with TD may actually reduce risk in these areas.

Despite these impressive research results there remains considerable skepticism regarding the utility of TTh in the medical community. The FDA has added to this skeptical attitude by minimizing the benefits of TTh in its assessment of the Testosterone Trials and by adding new restrictions for the indications of TTh. Although the FDA does not regulate the practice of medicine, it is widely regarded as a reliable authority, and its statements carry enormous weight, even when experts in the field disagree with the FDA's conclusions.

We believe the evidence presented in this study support the position that the importance of TD in health care and the benefits of TTh in these men is under-recognized and underappreciated.

We encourage HCPs to become more familiar with this literature. Greater awareness can be expected to lead to improved health and reduced health care costs for our society.

## Author Disclosure Statement

This article results from a symposium held on November 17, 2022, sponsored by Marius Pharmaceuticals. No author has received or will receive any financial compensation for participating, conceiving, writing, or editing of this article. The sponsor did not pay for the publication of this article, nor did it have any input or editorial control over the article's content.

## Funding Information

No funding was received for this article.

## Abbreviations Used

AF: atrial fibrillation
BAT: bipolar androgen therapy
CV: cardiovascular
FDA: U.S. Food and Drug Administration
HbA1c: glycated hemoglobin
HCPs: health care providers
HH: hypogonadotropic hypogonadism
MI: myocardial infarction
RWE: real-world evidence
T2DM: type 2 diabetes mellitus
T: testosterone
TD: testosterone deficiency
TTh: testosterone therapy
